# Lectures on Scarlet Fever

**Published:** 1851-05

**Authors:** Caspar Morris

**Affiliations:** Late Lecturer on Practical Medicine in the Philadelphia Medical Institute


### THE
### MEDICAL EXAMINER,
AND
### RECORD OF MEDICAL SCIENCE.

NEW SERIES.—NO. LXXVII.—MAY, 1851.

### ORIGINAL COMMUNICATIONS.

*Lectures on Scarlet Fever.* By Caspar Morris, M. D., late Lecturer on Practical Medicine in the Philadelphia Medical Institute.

### Lecture IV.

Though it be true that scarlet fever is in an especial degree a disease of infancy and childhood, all ages are, to a certain extent, liable to it. The reports of the Registrar-General of Great Britain are exceedingly interesting from the extensive field of observation they cover, and furnish us with the most important information on this and similar points. Thus, with regard to the point which now claims our notice, we find that during the months of January and February, 1840, there were 345 deaths from the disease, in London. Of this number 326 were under thirteen years of age, and 19 only were adults. And of 2614 deaths included in his fourth report for the Kingdom, 2429 were children, 182

adults, and 13 aged persons. Now, when we take into view the commonly conceded fact that the proportionate mortality of adults is much greater than of children, the real difference in liability will be found to exceed that which appears to be represented by these numbers, great as it is.

Dr. George Gregory says: "This is one of the few diseases to which the fœtus in utero is liable. On the 28th of April, 1839, my youngest son was born, evidently suffering under some form of fever. The throat was affected on the following day, obviously from angina maligna. Eruption was never developed. The child drooped and died on the first of May." As no attempt is made to show that the mother was laboring under the disease at or shortly before the birth of the infant, and it is not even asserted that it was prevalent in the neighborhood or family, there is great room to doubt the accuracy of the decision, that this was a case of scarlet fever. That persons at the other extreme of life may suffer from it I know, having seen it prove fatal in a lady, certainly not less than fifty years old.

Negroes are equally liable to this disease with whites, and dark as may be their hue, there is a peculiar change produced in it by the eruption where that is present, which is the result of the mingling of the florid tint with their peculiar color. When sudamina occur their appearance is very marked on the dark surface. The symptoms do not vary from those of the white, nor is the mortality greater.

Before we dismiss finally the consideration of the nature and laws of the specific cause of this disease, it is proper to draw your attention to the well established fact, that it is one of those to which we are liable but once. Dr. Willan, whose opportunities for observation were unusually great, and whose authority is beyond question, says that in more than two thousand cases which passed under his notice, he never met with a second attack. Dr. Tweedie asserts that there are well authenticated instances, but does not speak of them as of his own knowledge. No instance of a secondary attack occurred to Dr. Chapman, in his large and long extended observation. Dr. Currie, of Liverpool, was compelled by long experience to "renounce the opinion he had early entertained, and to confess that the same individual is liable to the disease once only." Sir

Gilbert Blane, on the other hand, asserts that he met with one instance of scarlet fever occurring thrice "without the least suspicion of ambiguity." Analogy favors the idea that in this, as in all other diseases, there are such exceptional cases. At an early period of my professional course, I thought I had met with them frequently myself. Longer and more careful observation has convinced me that, in many instances, I had mistaken other diseases for scarlet fever, misled by the similarity of the eruptions. There is a form of eruptive fever caused by indigestion, closely resembling this disease in its appearance, and like it making its invasion very suddenly, which is often met with in childhood. Such cases have undoubtedly been mistaken for scarlet fever, and have given rise to some of the reputed instances of second and third attacks. When occurring in the case of a child with enlarged tonsils, which are liable to acute inflammation from cold, or disordered stomach, the resemblance to scarlet fever is very close.

Secondary attacks may, however, occur, though less frequently in this than some of the other exanthematous fevers. A medical friend of great eminence has informed me that he lost a member of his own family by a second attack. The first was, when the child was but four years old, was irregular in its character, the eruption imperfect, and not followed by desquamation. There was an abscess in the neck, and long continued ill-health succeeded. After an interval of five years, the second and fatal attack supervened. There was but little affection of the throat, but the rash was very vivid and extensive, and death occurred from exhaustion of the vital forces by the intensity of the febrile reaction. Another medical friend has assured me that a well-marked second attack occurred in his family.

In this disease, as in variola and measles, second attacks are more frequent in those who have suffered from it in its most severe forms, than when in the first instance it has been mild. It would appear as though, in these persons, there was a greater degree of susceptibility to the influence of the contagious principle or epidemic miasm, while there are others in whom this liability exists but slightly at any period of life. This difference of susceptibility to the influence of the causes of disease, is one of those mysteries which it is but little likely we shall ever be able to solve. That of a given number of persons, equally exposed to an epidemic influence, only a small number will yield to the impression, is well known; and contagion itself finds the same exemption in kind, though not in degree. More extraordinary still, is the fact that this susceptibility varies in the same individual, at different times, without our being able to recognize the circumstances on which the fluctuation depends. Present exemption affords no security from future liability. Thus it can hardly be supposed probable that the two gentlemen who fell victims to it when more than forty years old, to whose cases I have already referred under another head, had never been exposed to the cause previously. About the same period, one of our most respectable physicians died from the disease. He had been many years engaged in a large practice, and must have encountered the complaint frequently, and been subject not only to the epidemic influence, but to direct contagion. In such cases the susceptibility must either have been recently developed, or some unknown power had caused it to lie dormant for years.

In this respect the laws of scarlet fever are perfectly consonant with those which govern the diffusion of other kindred diseases. Thus, Dr. Watson records an instance of fatal small-pox in the person of a lady beyond 70 years of age, who had nursed two generations of her descendants in the disease, and was therefore very naturally thought to possess an entire exemption from liability to it. There is a certain amount of susceptibility which is always present even in the persons of those who have formerly had the complaint, as is evidenced by the sore throat which affects those who are engaged in nursing persons sick with this disease. Such cases may indeed be considered as instances of secondary affections, especially as I have already proved that they are capable of propagating the disease. There is one fact connected with this branch of the subject which deserves to be impressed upon your minds, as it will naturally suggest important precautions. You will often find an error in diet, exposure to cold, or undue fatigue, prove the direct agent in exciting the disease. In such instances it is manifest that the specific cause was present in the system, but kept in subjection by the conservative force, until some accidental circumstance disturbed the balance of health, and afforded the opportunity for development which was needed. To caution persons against such exciting causes when the disease is prevalent in a neighborhood or has invaded the family circle, is, therefore, the duty of the physician.

The *mortality* of scarlet fever varies greatly, occurs at different periods of the progress of the case, and depends on various causes. The *prognosis* is therefore necessarily indefinite. So treacherous is the disease, and so liable to serious complications and fatal sequelæ, that it is best always to give a guarded opinion as to the result. You may say, with safety, that no case is so desperate in its symptoms as to shut out the hope of recovery ; but must at the same time admit that none is so benign as to exclude anxiety. I still remember perfectly a lesson learned in the earlier part of my professional career. I was attending the child of a clergyman ; of course, therefore, an object of attention for a whole congregation. The case was one of the mildest I had ever seen ; scarcely any anginose affection, the rash florid, the nervous centres unaffected. The progress of the disease was regular till the fourth day. It was a Sydenham case, "scarcely worthy the name of disease." Under these circumstances I ventured to congratulate the mother on her happiness. The disease was making fearful havoc in many families ; she would be exempt thenceforth from the apprehension in which she had participated with all parents. The case became complicated with laryngitis, and, forty-eight hours after, the child was a corpse. I have, on the other hand, seen a patient lie utterly unconscious of surrounding objects, with a livid eruption, entire loss of the power of deglutition, constant discharge of putrid sanies from the mouth and nostrils, and uncontrollable jactitation—and others with convulsions and croup,—and yet recover. You will not, therefore, be surprised when I tell you that my uniform habit is to speak cautiously of the lightest, and to encourage hope in the gravest case.

There is no doubt that the mortality among adults is greater in proportion than among children. To pregnant and puerperal women, it is almost inevitably fatal. I have known several cases which proved mortal but have never heard of a recovery. The character of the prevailing epidemic should always be taken into consideration in forming an estimate of the probable results of any case, as in no disease is the mortality more variable. This is proven by the personal experience of every practitioner. We may, however, refer to the report of the London Fever Hospital, given by Dr. Tweedie, as offering an illustration which cannot be questioned; 644 cases were treated in that institution during the twelve years from 1822 to 1833 inclusive. Of this number, thirty-eight died, being at about the rate of 6 per cent. upon the whole series of cases. But when we descend to the investigation of the annual mortality, we find that while, in 1829, the rate was as high as one in six, in 1832 it fell as low as one in forty. Dr. G. Gregory refers to the observations of a Mr. Ward of Bodmin, who reports four hundred and forty-two cases with thirty-six deaths; of these cases, three hundred and twenty-four were accompanied by the eruption, and of these, twenty-six died. While of one hundred and eight without eruption, ten died, thus making but a small difference in favor of those cases in which the skin is involved. Dr. Condie, of this city, reports two hundred and sixty cases, of children under 12 years of age, occurring during three epidemics of considerable extent and severity, with 45 fatal results. Dr. G. Gregory states that 6 per cent. may be assumed as the medium rate of mortality.

Though it be true, as I have before remarked, that the general character of the prevailing epidemic will throw light upon the probable result of the individual cases, it is right that you should know that there are exceptions to this rule. Not only do we find some cases of a very mild character occurring at the time, when the disease is generally malignant, but the reverse also takes place, though less frequently. You may adopt it as the general rule, that, whatever may be the prevailing type, the character of the first case which is seen in a family or school, will attach itself to all the subsequent cases. Thus the disease in the House of Refuge, was certainly very mild, no death having occurred, though the type of the epidemic, then prevailing in the city, was severe.

When, in forming your prognosis, you come to the consideration of the individual symptoms, I would caution you against placing any reliance on the extent of the eruption, as affording a criterion by which you may determine the degree of risk. I have frequently seen cases, in which the whole surface was covered with a vivid rash, prove fatal, and equally often have known the ready recovery of those in which there was none, or but little around the joints or on the body.

Simple scarlet fever is certainly much less likely to result fatally than the anginose, and this less so than the malignant and congestive, or irregular. The occurrence of convulsions in any stage, or either form, is always an indication of great danger. I at one time thought them a certainly fatal sign, but I have known several such cases to recover. Violent delirium, manifesting itself by shrieks like those of meningitis, is also of bad augury, no matter how slight may be the affection of the throat; so also is stupor with grinding of the teeth. Croup is a very serious complication and should cause you to watch the case in which it occurs, narrowly. The enlargement of the neck without a corresponding degree of swelling of the tonsils and adjacent mucous membrane, is also of evil omen; especially when there is the acrid discharge from the nostrils, which indicates the existence of ulceration of the posterior nares. The continuance or increase of the febrile symptoms, after the fifth or sixth day, is also a bad sign, as it proves the existence of some local lesion. A dusky or livid color of the eruption is always a bad sign, whether it result from the deficient aeration of the blood, or some other cause; and so are great sighing, and a feeling of faintishness, and hurried respiration. Ulceration of the commissures of the mouth and eyelids are unfavorable events, and so are coldness of the surface, whether it be dry, or bedewed with moisture.

The subsidence of the frequency of the pulse, and the diminution of the redness and swelling of the parts about the throat, in the anginose variety, are circumstances which should afford encouragement. Among the favorable signs, none should be hailed with greater satisfaction than a warm perspiration; whether it is found in the simple, anginose, or malignant form. Next to this is the diminution of the heat of the skin, with or without moisture, provided it do not descend to a degree of coldness less than the standard of health.

Death may result from various causes, and at several stages of the disease. The violence of the primary shock may be such that the system may not react from it, or the reaction may be so severe as to prove fatal. The nervous centres may be so deeply impressed that death shall ensue from the destruction of the balance necessary to their healthy action. From any of these causes it may occur within the first week of the disease; nay, even within the first twenty-four hours. Dr. G. Gregory thinks sixty hours, or the third day, the period of greatest danger. But even when the first week has passed, and the primary symptoms have yielded, the causes of mortality still stand thickly around the course of the patient. Inflammation of the larynx, the pericardium, or the kidneys, may be developed and produce a fatal result; or the patient may die from effusion in the ventricles of the brain or the thorax, or from the long continued exhausting effects of abscesses or diseases of the bones.

Each of these latter causes of death will of course leave a corresponding lesion in the organ affected. But in those cases where death has occurred in the early stages, it would be irrational to expect to find any morbid appearances in this, more than other pure febrile diseases. The fatal impression has been made on organs which express their changes by signs which pass away with life and leave no trace behind; but by some authors the brain is said to be more vascular than natural in cases where there has been very violent delirium, and instances are reported of increased vascularity and opacity of the arachnoid. We are not informed at what period of the disease death had occurred in these cases. In the malignant and anginose forms we shall of course find the traces of the disorganization of the mucous membrane of the fauces and adjacent parts which has been noticed during life, and I have seen ulceration of the mucous membrane lining the larynx, and abscesses about the cartilages.

Though there can be no doubt that the blood during life is much changed from its healthy condition, there is nothing peculiar in its state after death. It is reported to have been found sometimes fluid, at others abounding in coagula, sometimes collected in especial organs, and at others more equably distributed. The proportion of fibrine is said to be slightly in excess in some instances. This would result from any local inflammation which might be set up on the progress of the case. The spleen in some instances has been found enlarged, soft and reddened.

There are several *complications* which are met with in the different forms of scarlet fever, with which you should be made familiar. The sore throat in the anginose forms, varies in degree, and is often attended by swelling of the glands of the neck, impeding deglutition, even in some cases to the extent of entire interruption. It is by no means an uncommon event for the food and drink to be returned through the nostrils, instead of passing into the œsophagus. Ulceration of the mucous membrane, and even sloughs of the subjacent parts are found in extreme cases. Delirium is by no means an unfrequent attendant, and may be either wild and violent, or of a low muttering form. In the former case, which is confined to the simple and anginose varieties, it is frequently associated with convulsions; in the latter, which belongs to the malignant, with coma. Laryngitis is another serious complication which may occur in either form of the disease, and at any stage of its progress, and is always a formidable symptom. Violent rheumatic pains in the limbs are occasionally found in the earlier stages, as well as among the sequelæ, and there is often more or less muscular rigidity of the neck.

Ulcerations of the mucous membrane of the nasal passages, and the extension of the inflammation through the eustachian tube to the internal ear, are also met with in some cases, giving rise to the most intense suffering at the time, and involving the disorganization of the organ of hearing. Pneumonia *may* also occur in this disease, but more frequently violent affections of the heart indicated by labored respiration and distress in the præcordial region. Dr. Gregory describes the case of a lady "who was seized with scarlet fever at the time of parturition. The labor was long and severe; she perspired profusely; the heart labored violently. The next day scarlet fever appeared. The heart exhausted by previous efforts gave way, and in about fifteen hours from the appearance of the eruption, became engorged. A frightful feeling of suffocation supervened, and the pulse for a few minutes was imperceptible at the wrist. This feeling subsided, but the heart never regained its natural condition. Dyspnœa increased, and in twenty-four hours more, the blueness of countenance and incipient delirium showed that the lungs were implicated, and that waves of ill oxygenated blood were permeating the brain. Twelve hours longer of this semi-asphyxiate state closed this sad and painful scene."

The *Diagnosis* of scarlet fever is, you will perceive, not devoid of difficulty, and is yet always important. The anxiety of parents is excessive. Often, very often, have I been called at a late hour in the evening to see a child, suddenly seized with fever between the time at which it had been put to bed, and the hour at which the parents retired. The flushed cheek and restless sleep had excited the apprehension of a mother, some of whose friends had suffered from this devastator of the nursery. The power of allaying these fears by the positive assurance that no such danger was impending, is one of those which will afford you as much gratification as any you can exercise. Your own satisfaction in the treatment of a case is also much enhanced by certainty as to its character. Circumstances may also arise, in which it is most important to be able to give a prompt decision. I once, needlessly, caused the dismissal to their homes, of the children of a boarding school, thus spreading alarm into many families, and interrupting the pursuits and materially interfering with the interests of a most worthy family, by the hasty and erroneous determination that one of the children had scarlet fever. The opposite error might have been still more disastrous in its results. Let me, therefore, impress upon you the importance of a careful study of the signs of distinction. The well marked anginose and malignant forms can not be mistaken. It is only in the simple and irregular cases that we are liable to error. These, however, you remember, may give origin to the most violent and fatal forms, and even the mildest cases may become serious in the results, if not properly treated. In every case where there is a sudden invasion of fever with sick stomach and the occurrence of rash within forty-eight hours, you should *suspect* scarlet fever. Let this suspicion be your own, however, until you have carefully investigated the entire history of the attack, and examined well the symptoms, bearing in mind that important results may follow your decision.

There are several diseases which may be mistaken for simple or anginose scarlet fever. Measles, you know, was long confounded with it. Sore throat with fever resembles it in some points. The rash which often accompanies indigestion in children is very like it. There is a very mild form of disease, known popularly by the name of "the scarlet rash," which closely resembles it, and roseola has often been taken for it by superficial observers.

There are several points of difference between Measles and Scarlet Fever noticed by authors. The period of incubation is said to differ. When, however, you reflect on the impossibility of determining the time of exposure in many cases, and that when this is known, the mere fact of exposure will determine the nature of the case, you will at once perceive how little benefit can be derived from such an observation. I have, moreover, already shown how very uncertain is the period of incubation. It is quite certain, however, that measles has never been known to occur so promptly after exposure as scarlet fever.

The time, after the development of febrile symptoms, at which the eruption occurs in the two diseases in those cases which pursue a regular course, is very different. The rash of scarlet fever is observed often simultaneously with the febrile invasion, and generally within twenty-four hours; that of measles does not show itself in less than seventy-two hours, or on the third or fourth day. The invasion of scarlet fever is marked by nausea or vomiting, thus fixing the stomach, and passages which lead to it, as the organs involved. The first stage of measles, on the other hand, is indicated by catarrh, sneezing, redness of the conjunctiva cough and hoarseness; thus exhibiting the tendency of the disease to fix on the mucous membrane of the air passages. This difference in the local tendencies marks the whole course of the disease and its sequelæ.

There is also a marked difference in the appearance of the rash. The eruption of measles may be first seen about the roots of the hair and behind the ears; in circumscribed well defined spots, which as they spread, assume frequently an arrangement which has been called crescentic, several spots being grouped together and surrounding patches of skin of perfectly natural appearance. From the face, it gradually extends itself downward over the whole person. In the regular course of scarlet fever, there is a vivid efflorescence covering the whole surface of the head and neck simultaneously, and often even the whole trunk, leaving no spot unaffected. The papular eruption scattered through the efflorescence of scarlet fever, which gives the appearance of stigmata, noticed in the description of the disease, is unknown in measles. There is a marked difference also in the color of the rash of the two diseases. In scarlet fever it is of a florid or vermillion tint; in measles there is at the very outset a dull lake color. The first declines by fading away; the last becomes darker or more dusky.

In the sequelæ of the two diseases, we find the most striking evidence of their diverse origin and nature.

In scarlet fever we have affections of the brain, the kidneys, the cellular tissue, ulcerations of the throat and ears, and abscesses about the neck, rheumatic pains, and if any of the affections of the air passages occur, it is evidently by mere extension from the adjacent parts. In measles we have bronchitis and pneumonia, and sometimes diarrhœa. But while there are these important and well marked points of difference between the two diseases, it must not be concealed that you will occasionally meet with instances in which your efforts at discrimination will be tasked during the progress of the case; and it is even possible that in some there may be a mongrel disease, as though it were the result of a mixed influence; commencing as scarlet fever and yielding in its course to the influence of rubeola. There is often in cases of variola or varioloid a diffuse efflorescence on the second or third day which very closely resembles that of scarlet fever, and the resemblance is rendered more embarrassing by the sore throat which so generally occurs in variola. It may be distinguished from it by the character of the febrile symptoms which accompany it. The restlessness and gastric oppression and pain in the back, of the variolous disease, sufficiently mark its character, and are never present in scarlet fever. Neither is there the frequent pulse to which I have drawn your attention, as belonging especially to the disease. That of the variolous impression is less frequent and softer, the absence of the dotted points which are interspersed through the efflorescence on the skin, and always appear in the throat, will afford assistance in determining the nature of the case. Attention to the symptoms affords one of the best points of discrimination in cases of eruption resembling scarlet fever which occur in the signs of indigestion in childhood and infancy, and the best additional diagnostic sign I can furnish you is found by looking into the throat. The eruption of scarlet fever is *always* found there, if it affect the external surface. The appearance of the elongated papillæ through the white fur, is less to be relied on. I have been deceived by it. Not so, however, the appearance of the tongue on the second or third day. The sudden throwing off of the coat of fur, while the surface is left red and glossy, is decidedly characteristic and will never deceive you, as it occurs in no other disease at this early period.

The eruption which occurs about the third day of typhus fever, bears a closer resemblance to that of measles than to the one now under our notice. The age of the patient will afford aid in forming an opinion, typhus fever being more frequent in adult life, and scarlet fever in infancy and childhood, while the history of the case will always enable you to trace genuine typhus fever to its origin in direct contagion or exposure to the peculiar circumstances which give rise to it.

(To be Continued.)

